# The mouse mammary gland as a sentinel organ: distinguishing ‘control’ populations with diverse environmental histories

**DOI:** 10.1186/s12940-017-0229-1

**Published:** 2017-03-09

**Authors:** SriDurgaDevi Kolla, Aastha Pokharel, Laura N. Vandenberg

**Affiliations:** 1 School of Public Health and Health Sciences, University of Massachusetts, Amherst, USA; 2 School of Public Health and Health Sciences, University of Massachusetts, Amherst, USA; 3 Department of Environmental Health Sciences, School of Public Health and Health Sciences, University of Massachusetts, 171A Goessmann, 686 N. Pleasant Street, Amherst, MA 01003 USA

**Keywords:** Endocrine disruptor, Estradiol, Volumetric morphometric historical control, Contamination, Sensitivity, Guideline endpoint, Commercial supplier

## Abstract

**Background:**

There are numerous examples of laboratory animals that were inadvertently exposed to endocrine disrupting chemicals (EDCs) during the process of conducting experiments. Controlling contaminations in the laboratory is challenging, especially when their source is unknown. Unfortunately, EDC contaminations can interfere with the interpretation of data during toxicological evaluations. We propose that the male CD-1 mouse mammary gland is a sensitive bioassay to evaluate the inadvertent contamination of animal colonies.

**Methods:**

We evaluated mammary glands collected from two CD-1 mouse populations with distinct environmental histories. Population 1 was born and raised in a commercial laboratory with unknown EDC exposures; Population 2 was the second generation raised in an animal facility with limited exposures to xenoestrogens from caging, feed, etc. Mammary glands were collected from all animals and evaluated using morphometric techniques to quantify morphological characteristics of the mammary gland.

**Results:**

Population 1 (with suspected history of environmental chemical exposure) and Population 2 (with known limited history of xenoestrogen exposure) were morphologically distinguishable in adult males, prepubertal females, and pubertal females. Mammary glands from males raised in the commercial animal facility were significantly more developed, with larger ductal trees and more branching points. The appearance of these mammary glands was consistent with prior reports of male mice exposed to low doses of bisphenol A (BPA) during early development. In females, the two populations were morphologically distinct at both prepuberty and puberty, with the most striking differences observed in the number, size, and density of terminal end buds, e.g. highly proliferative structures found in the developing mammary gland.

**Conclusions:**

Collectively, these results suggest that the mouse mammary gland has the potential to be used as a sentinel organ to evaluate and distinguish animal colonies raised in different environmental conditions including potential EDC exposures. Our findings could help researchers that wish to perform *a posteriori* evaluations to determine whether inadvertent contamination with xenoestrogens (and potentially other EDCs) has occurred in their animal colonies, especially after new materials (feed, caging, water bottles) have been introduced. Finally, our results challenge the relatively common practice of using historical controls in toxicological experiments.

## Background

Endocrine disrupting chemicals (EDCs) are compounds, or mixtures of compounds, that interfere with hormone actions by altering hormone synthesis, secretion, binding, metabolism, or elimination [1, 2]. Scientists that study EDCs in controlled laboratory experiments must take concerted efforts to control contaminations from environmental chemicals that could interfere with the assays being conducted and/or the endpoints being evaluated [3, 4]. These contaminations can be introduced by standard laboratory consumables (disposable plastics, storage containers), disinfectants, laboratory equipment, and other sources. In vitro assays designed to examine the role of estrogens in breast cell proliferation were some of the first studies to show that xenoestrogens can leach from commercially available plastics [5, 6]. Further, studies in mice that were originally intended to evaluate age-dependent changes in ovarian function and the timing of pubertal onset were disrupted by the unanticipated leaching of estrogenic chemicals from animals’ caging materials [7–10].

Experiments using laboratory animals must consider a number of different potential sources of EDCs including caging materials, water bottles (or tubing to deliver water directly to the cage), bedding, enrichment materials, food, water, and even the air in the animal facility [3, 11, 12]. A failure to rule out contamination can make it difficult, or impossible, to draw accurate conclusions about the endocrine disrupting properties of test chemicals. For example, if an experimenter wants to evaluate whether a test chemical displays estrogenic properties but all animals (including the unexposed negative controls) are inadvertently exposed to a xenoestrogen from the environment, the treated animals may appear to be non-responsive due to maximal stimulation of all animals [13, 14].

Significant efforts have been invested to identify the most sensitive endpoints to reveal the effects of EDCs [15, 16]. Whereas traditional endpoints of toxicity (e.g. significant loss of body weight, altered organ weight) are typically used to evaluate chemicals for regulatory purposes, numerous studies have demonstrated that the endpoints evaluated in test guidelines are not sufficiently sensitive to predict the effects of chemicals on humans, particularly when exposures occur during vulnerable periods of development [17–19]. Studies that have directly compared the effects of traditional guideline endpoints with non-guideline endpoints are relatively rare [20], but a large volume of data suggests that non-guideline endpoints can identify adverse effects of EDCs at doses far below those that alter organ weight [21–23].

Our own work, and work from multiple other laboratories, has shown that the rodent mammary gland is sensitive to EDCs, especially when exposures occur during critical windows of development [24–28]. We recently showed that male CD-1 mice exposed to bisphenol A (BPA), a well-known xenoestrogen, during the perinatal period develop larger and more elaborated mammary epithelial trees in adulthood [29]. These findings were particularly interesting because male mice do not have nipples, leading some researchers to conclude that they do not retain any epithelial tissues [24]. The male CD-1 mouse mammary gland may in fact be a sensitive bioassay that could be used to evaluate novel compounds as well as evaluate the potential for inadvertent contamination of animal populations with xenoestrogens, and perhaps compounds with other endocrine disrupting properties [30, 31].

While conducting controlled laboratory experiments, we have collected mammary glands from adult male CD-1 mice that were born and raised *in a commercial laboratory*. We noted that the epithelial trees in these males were much larger and more developed than what we previously reported for ‘control’ adult male mice; rather, these males had mammary tissues that more closely resembled animals developmentally exposed to BPA [29]. Based on those observations, we asked if the morphology of the mouse mammary gland can be used to distinguish colonies of mice raised in different environments including groups that *may* have experienced EDC exposures. Here, we report the results of our evaluations of two populations of mice: CD-1 mice ordered from commercial laboratories, and CD-1 mice raised in our animal facility for two generations under controlled conditions with limited environmental chemical exposures (e.g. exposures that were minimized wherever possible). We find that these populations are distinguishable, with the most striking differences in the adult male mammary glands, suggesting this may be an appropriate sentinel organ to evaluate an animal’s prior environmental exposure to xenoestrogens and other EDCs.

## Methods

### Animals

Two populations of animals were evaluated in this study. *Population 1:* CD-1 mice were ordered from a commercial supplier (Charles River Laboratories, Raleigh, NC) in adulthood (males, approximately 8 weeks of age) or at postnatal day (PND) 22 (females, prepubertal) or PND32 (females, pubertal). Upon arrival, these animals (referred to in the text as CRL animals) were group housed (typically 4 animals per cage) in polysulfone cages. Animals were provided a low phytoestrogen feed (Harlan Teklad 2018) which has previously been shown to have estrogenic activity in the low femtomolar range [32] and tap water in glass bottles. CRL animals were housed under these conditions for 2 to 3 weeks (adult males), 2 days (prepubertal females) or 3 days (pubertal females). The males were also used as breeders for other experiments.

#### Population 2

Timed pregnant female CD-1 mice (pregnancy day 4–5) were ordered from Charles River Laboratories and housed according to the details provided above for *Population 1*. These dams were allowed to deliver naturally, and their litters (the F1 generation) were culled to 10 pups on PND1. Litters were weaned on PND21, the female F1 offspring were group housed with up to 2 littermates and raised to adulthood (approx. week 8). One F1 female from each litter was mated to a control male (ordered from Charles River Laboratories), and these females were also allowed to deliver naturally. Litters were culled and weaned in the same manner as the previous generation. The F2 offspring were continuously housed under controlled conditions until they reached the same ages as in *Population 1.* These animals will be referred to throughout the text as F2 animals.

All animals were maintained in temperature and light controlled (12 h light, 12 h dark, lights on at 0800 h) conditions at the University of Massachusetts, Amherst Central Animal Facility. All experimental procedures were approved by the University of Massachusetts Institutional Animal Care and Use Committee (protocol 2014–0055).

### Necropsies and dissections

At all ages (PND24, PND35, and adulthood), mice were killed via CO_2_ inhalation followed by decapitation. Blood was collected from all animals. The right fourth inguinal mammary gland was isolated using standard dissection methods, spread on a positively charged clear slide (Fisher, Waltham MA), and fixed overnight in neutral buffered formalin (Fisher) at room temperature.

### Carmine alum whole mount processing/staining

Following fixation, whole mount mammary glands were washed, dehydrated through an alcohol series and defatted with toluene (Sigma-Aldrich, St. Louis MO). The glands were then stained overnight with carmine alum (Sigma-Aldrich), dehydrated through a series of alcohols and xylene, and then placed in k-pax heat-sealed bags (Fisher) with methyl salicylate (Fisher) to preserve them.

### Mammary gland morphometrics

Whole mounts mammary glands were viewed and imaged using a Zeiss Axio Imager dissection microscope and Zeiss high-resolution color camera (Carl Zeiss, Oberkochen, Germany). Zeiss ZEN Pro software was used for morphometric analysis using methods developed previously [29, 33]. Briefly, in females at both ages, specific measurements were quantified including the area subtended by the ducts (ductal area), the growth of the longest duct from the center of the lymph node (ductal extension), the total number of terminal end buds (TEBs, defined as bulb-shaped structures ≥0.03 mm^2^), and area of TEBs. Ductal density was evaluated using the threshold tool to compare intensity of mammary epithelium within 1.5 cm^2^ areas in the nipple and lateral to the lymph node, or across the entire ductal tree. In males, endpoints that were measured included ductal area and number of branching points. TEBs were never observed in adult males.

### Enzyme Linked Immunosorbent Assay (ELISA)

Serum was isolated from blood samples collected from all animals at the time of necropsy. A commercially available kit (Calbiotech, Cat# ES180S-100) was used to quantify 17β-estradiol levels in these serum samples. The absorbance of the samples was measured at 450 nm using a SpectroMax plate reader (Molecular Devices, Sunnyvale CA). The limit of detection using this kit was 1 pg/ml. Some samples from each group (CRL and F2) at each age (prepubertal and pubertal females, adult males) were run in duplicate, but many samples did not have sufficient serum to do this. Of those samples that were run in duplicate (8 samples total), the coefficient of variation ranged from 12 to 26.8%. For the calculation of average serum 17β-estradiol concentrations, animals with concentrations below the limit of detection were assigned a value of 0.1 pg/ml.

### Statistics

All experiments and measurements were conducted by experimenters that were blind to treatment group. Data were analyzed using SPSS version 23 (IBM Analytics, Armonk NY). For comparisons of CRL versus F2 animals in each age group, independent samples T-tests were used. An *a priori p*-value of 0.05 was selected for statistical significance. All data displayed in graphs represent mean ± SEM. Sample sizes for analyses are indicated in Table 1.

**Table 1:** Sample sizes evaluated in this study

	CRL (Population 1)	F2 (Population 2)
Adult males	22	16
Prepubertal females	15	24
Pubertal females	20	22

## Results

### The male mammary gland is morphologically distinct in two adult populations of CD-1 mice

**Fig. 1:**
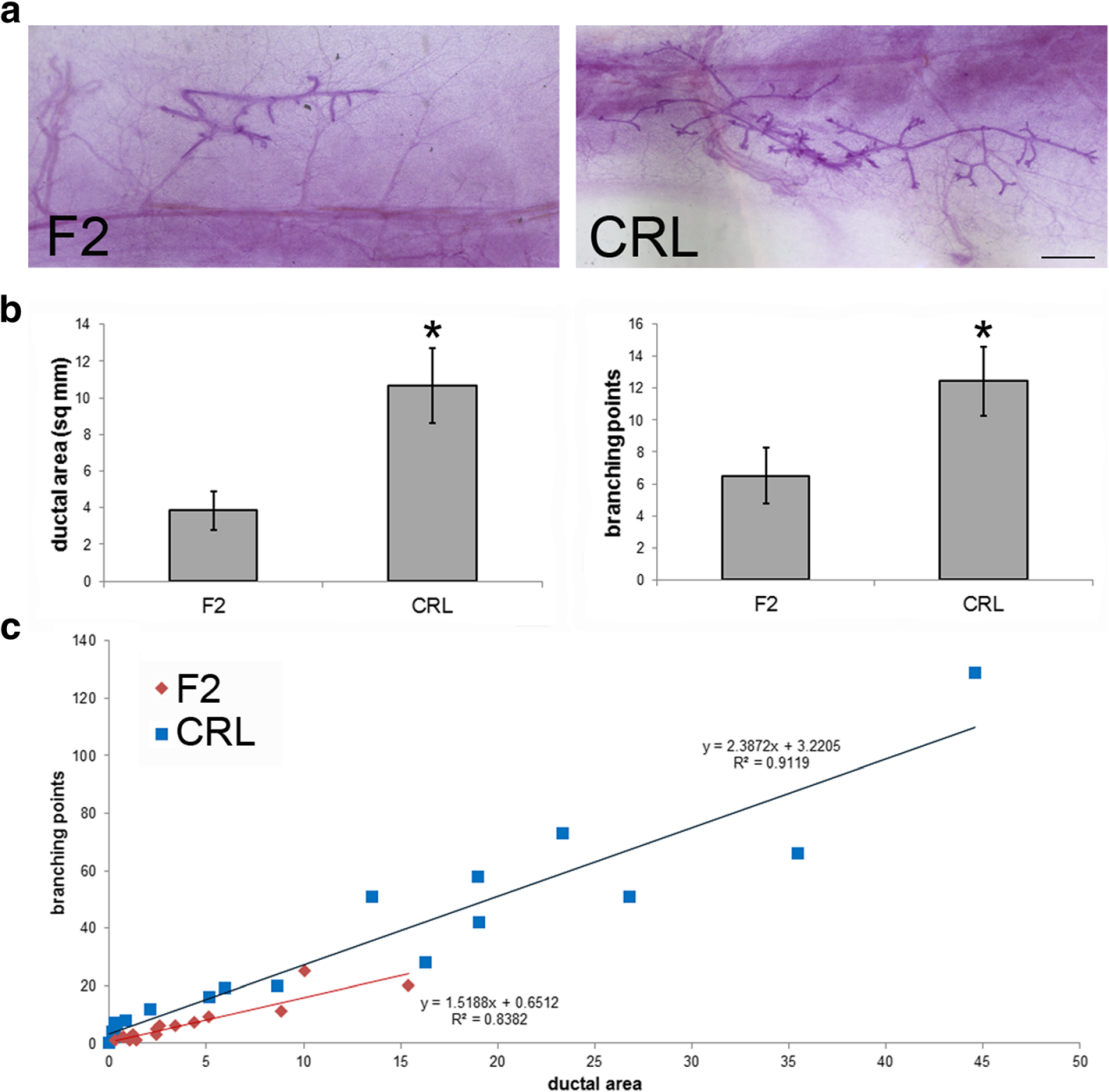
The mammary gland is larger and more developed in CRL adult males. **a** Representative whole mount mammary glands from F2 and CRL males. Scale bar = 1 mm. **b** Quantification of two parameters of the male mammary gland, ductal area (*left*) and number of branching points (*right*) reveals significant differences between F2 and CRL males. For both graphs, * indicates p ≤ 0.01. **c** Linear regression analyses reveal strong associations between the number of branching points and ductal area in both F2 and CRL males, but CRL males had more branching points per square millimeter of ductal area than F2 males

This project was initiated after we made observations suggesting that control, untreated male CD-1 mice from commercially available sources had larger epithelial trees than have been measured in untreated male CD-1 mice in previous studies [29]. To characterize and quantify the mammary gland morphology from CRL and F2 male mice, we evaluated two growth parameters in each gland: ductal area and number of branching points. For both parameters, the CRL animals were significantly more developed (Fig. 1a and b). Correlative analyses suggested that both populations displayed strong associations between the number of branching points and ductal area (Fig. 1c), consistent with observations that larger glands are also more elaborated (e.g. have more individual ducts). In the CRL males, the R^2^ value is 0.9119 (*p* < 0.001) and in the F2 males, the R^2^ value is 0.8382 (*p* < 0.001). However, statistical comparisons of these linear regressions did not reveal statistically significant responses between the responses observed in the CRL and F2 males (ANOVA, *p* = 0.106). Collectively, these results suggest that the CRL and F2 males are morphologically distinct because the CRL males have larger, more highly branched mammary glands compared to the F2 males, but that the relationship between ductal area and branching points is indistinguishable between CRL and F2 males.

### The number and size of TEBs are different between two pre-pubertal populations of CD-1 female mice

**Fig. 2:**
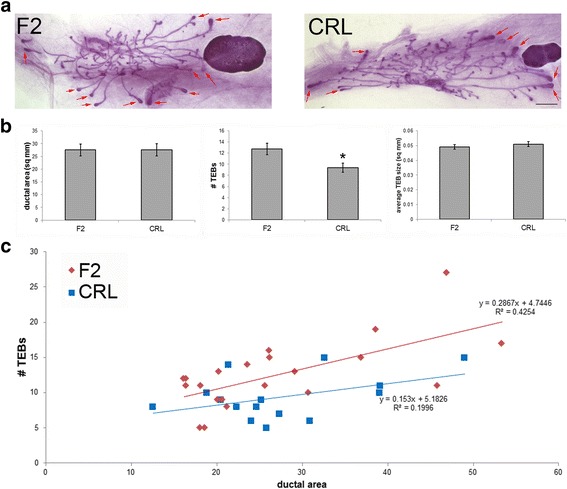
CRL females have fewer TEBs compared to F2 females at PND24. **a** Representative whole mount mammary glands from F2 and CRL females at PND24. Scale bar = 1 mm. Examples of TEBs are indicated by red arrows. **b** Quantification of three parameters of the female mammary gland, ductal area (*left*), number of TEBs (*middle*), and average TEB size (*right*). CRL females had fewer TEBs but no differences in TEB size or ductal area compared to F2 females. *, *p* < 0.05. **c** Linear regression analyses reveal positive associations between ductal area and number of TEBs in both F2 and CRL females. F2 females typically had more TEBs per square millimeter of ductal area compared to CRL females

We next examined mammary gland morphology in CRL and F2 females at PND24. This period of time was selected because it is prior to the onset of first proestrus and most animals do not yet display vaginal opening, two measures that are indicative of the onset of puberty [34, 35]. The area subtended by ducts was very similar between these two groups, but the number and density of TEBs was significantly lower in the CRL females compared to the F2 females (Fig. 2a and b and data not shown). Average size of TEBs was similar between the two groups (Fig. 2b). Using regression analysis, both populations displayed positive associations between ductal area and number of TEBs, suggesting that larger glands are also more developed (Fig. 2c). This association was only significant in the F2 females [CRL females, R^2^ = 0.1996, *p* = 0.095; F2 females, R^2^ = 0.4254, *p* < 0.001]. Statistical comparisons of these linear regressions did not reveal statistically significant differences between the relationship between ductal area and TEBs observed in the CRL and F2 females at prepuberty (ANOVA, *p* = 0.154). Taken collectively, these results suggest subtle but statistically significant differences between CRL and F2 females in prepuberty; these modest changes in morphology may be too slight to easily differentiate these populations.

### Several morphological features distinguish CRL and F2 females at puberty

**Fig. 3:**
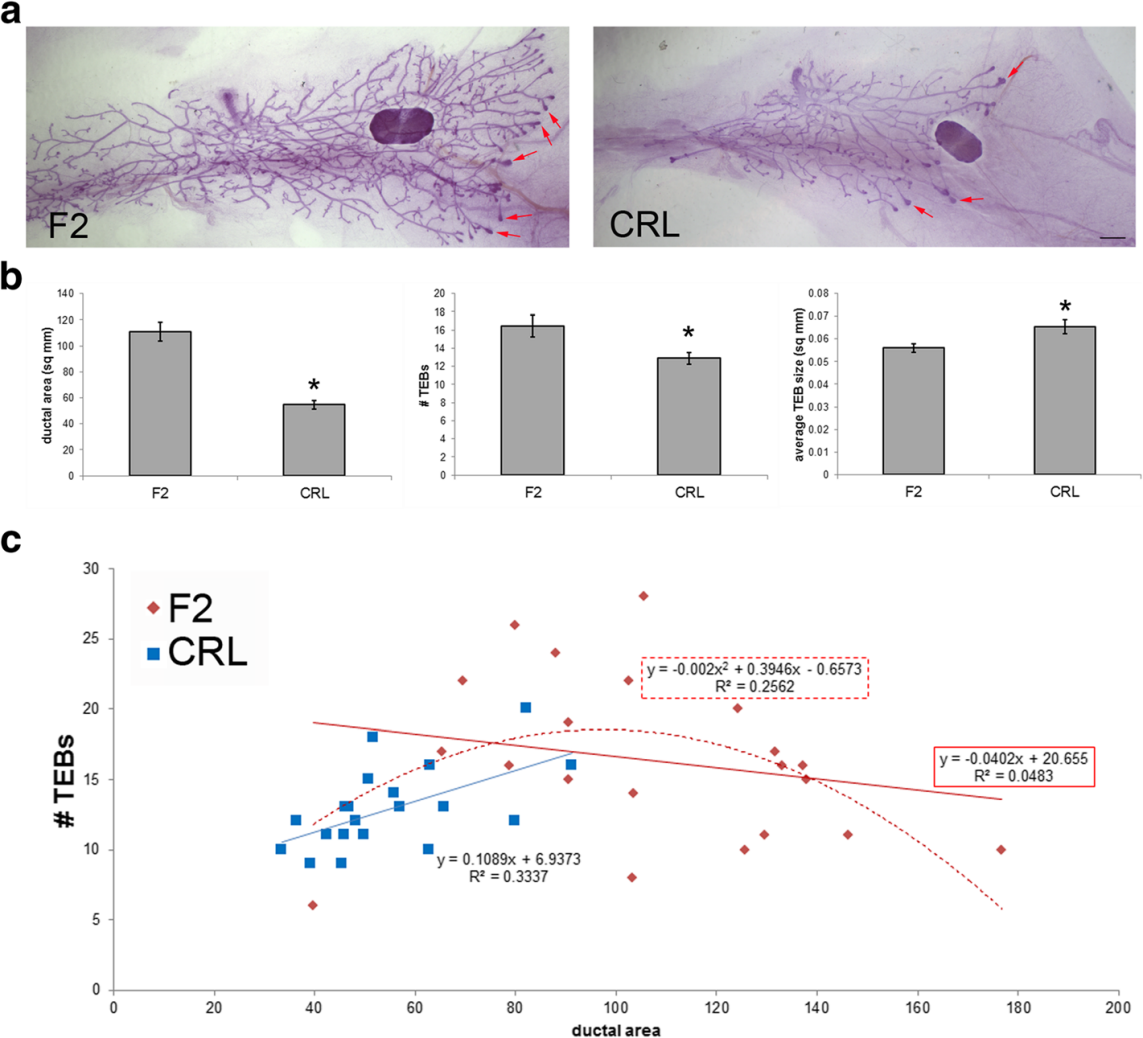
CRL females have smaller mammary glands with more dense TEBs at puberty. **a** Representative whole mount mammary glands from F2 and CRL females at puberty. Scale bar = 1 mm. Examples of TEBs are indicated by red arrows. **b** Quantification of three parameters of the female mammary gland, ductal area (*left*), number of TEBs (*middle*), and average TEB size (*right*). CRL females had smaller ductal trees, and fewer but larger TEBs compared to F2 females. *, *p* < 0.02. C) Linear regression analyses reveal positive associations between ductal area and number of TEBs in CRL females. However, there was little association between ductal area and number of TEBs in F2 females. For this reason, a polynomial regression was also performed (*see dotted line*), and this provided a much better fit for the CRL pubertal female data﻿

The third group of animals we evaluated included CRL and F2 females collected on PND35, after the onset of puberty. At this age, mammary glands from CRL females were significantly smaller compared to F2 females, with ductal areas approximately half as large (Fig. 3a and b). Total TEB number, TEB density and average TEB size were also significantly different between these groups, with more TEBs in the F2 females and a higher density and average TEB size in the CRL females (Fig. 3b and data not shown). We also observed differences in the density of the mammary epithelium in F2 and CRL pubertal females. The F2 females had significantly denser epithelium in the region adjacent to the nipple and in the entire ductal tree, but not in the lateral areas closest to the lymph node (Table 2). These results are consistent with increased amounts of internal branches and buds in the F2 females.

**Table 2:** Epithelial density in CRL and F2 females at puberty

	CRL (Population 1)	F2 (Population 2)	*p*-value (independent samples t-test)
Nipple region	0.518 ± 0.025	0.617 ± 0.027	*p* = 0.010
Lateral to lymph node	0.580 ± 0.026	0.639 ± 0.034	*p* = 0.178
Whole ductal tree	0.572 ± 0.015	0.660 ± 0.022	*p* = 0.002

Data provided represent means ± SEM, intensity is a unitless measure

Strikingly, when we evaluated the relationship between ductal area and number of TEBs using linear regression analysis, there was a statistically significant, positive association in the CRL animals (R^2^ = 0.3337, *p* < 0.01), but a null - or slightly negative - association in the F2 group that was not statistically significant (R^2^ = 0.0483, *p* = 0.493) (Fig. 3c). Comparing these linear regressions revealed the possibility that CRL and F2 females respond differently because there was a trend toward statistically significant differences between the responses observed in the CRL and F2 females at puberty (ANOVA, *p* = 0.075). Importantly, the relationship between ductal area and the number of TEBs appeared to be biphasic (non-monotonic) in the F2 pubertal females. A polynomial regression [order = 2] was a better fit for these data (R^2^ = 0.2562, *p* < 0.05, Fig. 3c). Together, these results suggest that these two mouse populations have different mammary gland appearances that allow them to be distinguishable.

### Serum estradiol concentrations do not distinguish CRL and F2 animals

**Fig. 4:**
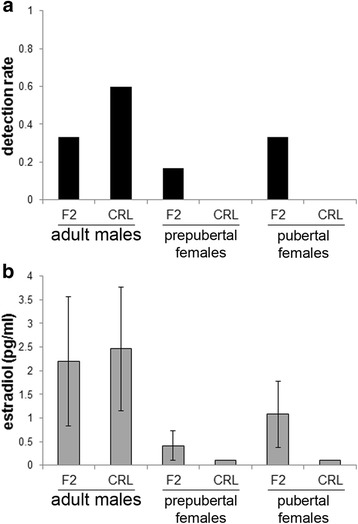
Serum 17β-estradiol levels are similar between F2 and CRL animals. **a** An ELISA kit with a limit of detection of 1 pg/ml was used. Detection rates ranged from 0 to 0.6 in each group. No significant differences in detection rate were observed for F2 and CRL animals in each sex/age group. **b** Serum 17β-estradiol levels were highest in adult males, but were also variable. Significant differences in serum concentrations were not observed between F2 and CRL animals for any sex/age group. Sample sizes for ELISA were *n* = 6 F2 males, *n* = 5 CRL males, *n* = 6 F2 prepubertal females, *n* = 5 CRL prepubertal females, *n* = 6 F2 pubertal females, *n* = 5 CRL pubertal females

To determine whether the differences in mammary gland development could be attributed to differences in circulating concentrations of 17β-estradiol, the hormone that drives ductal growth and TEB development, we evaluated serum concentrations of 17β-estradiol in adult males, pre-pubertal females, and pubertal females from the CRL and F2 groups. The detection rate was below 0.5 in all groups except the CRL males; no samples had concentrations above the limit of detection in CRL females at pre-puberty or puberty (Fig. 4a). When we compared concentrations in F2 and CRL animals, there were no significant differences in any of the groups (Fig. 4b). Collectively, these results suggest that serum concentrations of 17β-estradiol are not sufficient to distinguish populations of mice that have morphologically distinct mammary glands.

## Discussion

Our initial observations of male CRL mammary glands were that these glands were larger than expected, consistent with developmental exposures to EDCs like BPA [29]. In fact, the mammary glands collected from CRL animals were most similar to CD-1 male mice from prior studies that were developmentally exposed to BPA in the range of 2.5 – 25 μg/kg/day [29]. Further quantifications revealed that our F2 animals, raised in controlled conditions with concerted efforts to limit EDC exposures, display small ductal trees similar to what have been reported previously in other controlled environments [29–31, 36].

At first glance, the female data show that mammary glands from CRL females are smaller at puberty, which might be interpreted as suggesting that their chemical exposures are lower than those experienced in the F2 females. However, studies examining the effects of BPA on the pubertal mammary gland have shown that lower doses can increase ductal area whereas higher doses can decrease ductal area [35, 37]. These studies also demonstrate that BPA doses in the ng/kg and μg/kg range can significantly shift the density of TEBs in the mammary gland, to produce densities consistent with what we have observed in the CRL females. Thus, it is highly likely that all of the CRL animals experienced developmental exposures to xenoestrogens.

Is it possible that the CRL and F2 animals have some other feature that distinguishes these two populations, e.g. could our observations be due to genetic drift? Non-environmental differences between CRL and F2 animals seem highly unlikely, especially considering that all of our F2 animals were derived from animals that were ordered from the commercial supplier (e.g. F2 animals are only two generations removed from CRL animals), and it is unexpected that such drastic genetic changes could occur in just two generations of controlled breeding. However, our study is limited because we do not know what chemicals CRL animals might have been exposed to during development or the specific source of chemical exposures. Although our results are both qualitatively and quantitatively consistent with exposures to BPA in the low dose range [38], similar effects on the mammary gland are expected from exposures to other xenoestrogens including common chemical contaminants in animal feed, phytoestrogens, or other compounds that might leach from plastics used in cages, water bottles, or other housing devices [39–41]. Furthermore, our studies suggest that commercial laboratories are raising animals in environments that are not well defined when it comes to EDC exposures. We expect that these exposures are low enough that they are not affecting fertility or fecundity, as commercial animal suppliers are acutely aware of these health outcomes; our results here suggest that xenoestrogen exposures in vendor laboratories that have modest (or perhaps no) effect on reproductive outcomes could still influence other hormone-sensitive endpoints including the mammary gland.

It is theoretically possible to determine what chemicals the CRL animals were exposed to during early development. First, some chemicals with longer half-lives may still be present in the blood of these animals at the time of necropsy, or additional animals could be sacrificed immediately upon arrival for the collection of blood and tissues. It is important to note that collecting serum for these purposes can be difficult because of the potential for contamination, the requirement for large volumes of serum to do broad screening rather than measures for single compounds, and concerns that the most relevant exposures likely occurred during embryonic and fetal development and thus may no longer be present in the blood of animals that are weeks or months old [42–44]. Recent studies have shown that novel metabolomics methods can reveal persistent changes in metabolic “fingerprints” including variations in glucose, amino acids, and neurotransmitters in the serum of animals exposed to BPA during early development [45]. Altered metabolomic profiles were maintained for at least 180 days after exposures ceased [46]. These studies suggest the possibility that features of an animals’ serum could be used to not only determine what compound(s) they were exposed to, but also what dose. However, until such metabolomics profiles are available for the most common environmental contaminants, their use in the retrospective determination of chemical exposures will remain very limited.

Certainly, *a priori* screening of items in the animal facility is the best approach to avoid environmental chemical contamination of animals to be used in laboratory experiments. However, the ability to perform *a posteriori* evaluations to determine whether inadvertent contamination has occurred remains important, especially when new materials are introduced in animal care settings without the knowledge of the experimenter. In these cases, post hoc evaluative approaches using metabolomics are very resource intensive and require extensive training in sensitive molecular biology techniques. In contrast, analysis of mammary gland morphology is straightforward and inexpensive. For this reason, the tools we have demonstrated in this manuscript may prove to be sufficient to distinguish populations with distinct environmental histories that include chemical exposures during vulnerable periods of development.

There are two important consequences of our findings which are consistent with low-level xenoestrogen contamination in commercially available animals. The first arises from the use of adult animals for toxicity screening of environmental chemicals. If an experimental protocol requires that adult animals be exposed to test compounds, developmental exposures to EDC mixtures could interfere with the accuracy of this testing. There are a number of studies that have demonstrated that early life exposure to BPA alters responsiveness of animals to hormones or carcinogen challenges at puberty or in adulthood [35, 47–52]. This “two hit” model suggests that, for some endpoints, developmental exposures may not be sufficient to induce adverse effects, but the consequences of EDC exposures are best seen when a secondary challenge is experienced [22]. In this case, the uncontrolled EDC exposures that occur in commercial animal facilities could represent “hit one” and the controlled exposure experienced during toxicity testing would actually be “hit two”.

The second concern related to uncontrolled EDC exposures is the use of historical controls in toxicity testing in commercial laboratories. In some testing protocols, when the negative controls have unexpected responses, experimenters will instead use “historical controls”, e.g. unexposed controls collected in previous experiments, including experiments that may have been conducted years prior [53]. A report from the Historical Control Working Group from the Society of Toxicologic Pathology noted that “[s]tudy design-related parameters such as laboratory, species/strain, route of administration, vehicle, feed, feeding practices, study duration, and housing have a potential to impact study outcomes and control findings. These parameters should be considered when selecting the appropriate studies” to generate historical control data [54]. Yet, differences in background EDC exposures may occur even when experimenters think they are following the guidance of this working group. Other groups have highlighted differences in endocrine endpoints in historical controls from different testing facilities used to evaluate chemicals for endocrine disrupting activities [55, 56], suggesting that this issue may be more widespread than the single commercial laboratory we examined. Our results suggest that ‘control’ animals may not be unexposed; thus, using historical controls which might have one set of background EDC exposures to compare to experimental animals with an entirely different environmental history should not be acceptable practice [11].

## Conclusions

Here, we compared mammary gland morphologies for two “control” populations with distinct environmental histories and found significant differences in adult males, prepubertal females, and pubertal females. These results suggest that the mammary gland may be a sensitive organ to probe populations for unknown environmental EDC exposures during vulnerable periods of development. Our results also dispute the use of historical controls because environmental EDC exposures may differ between experiments due to changes in cages, water bottles, animal feed, water, or other environmental factors that might otherwise appear benign.

## Abbreviations

BPA: Bisphenol A
CRL: Charles River Laboratories, Population 1
EDC: Endocrine disrupting chemical
ELISA: Enzyme linked immunosorbent assay
F2: F2, The second generation raised under controlled conditions, Population 2
PND: Postnatal day
TEB: Terminal end bud
